# Anti-Obesity Effect of a Tea Mixture Nano-Formulation on Rats Occurs via the Upregulation of AMP-Activated Protein Kinase/Sirtuin-1/Glucose Transporter Type 4 and Peroxisome Proliferator-Activated Receptor Gamma Pathways

**DOI:** 10.3390/metabo13070871

**Published:** 2023-07-21

**Authors:** Mohamed A. Salem, Nora M. Aborehab, Mai M. Abdelhafez, Sameh H. Ismail, Nadine W. Maurice, May A. Azzam, Saleh Alseekh, Alisdair R. Fernie, Maha M. Salama, Shahira M. Ezzat

**Affiliations:** 1 Department of Pharmacognosy and Natural Products, Faculty of Pharmacy, Menoufia University, Gamal Abd El Nasr Street, Shibin Elkom 32511, Menoufia, Egypt; mohamed.salem@phrm.menofia.edu.eg; 2 Department of Biochemistry, Faculty of Pharmacy, October University for Modern Sciences and Arts (MSA), Giza 12451, Egypt; naborehab@msa.edu.eg; 3 Department of Pharmacology and Toxicology, Faculty of Pharmacy, October University for Modern Sciences and Arts (MSA), Giza 12451, Egypt; maimoustafa@msa.edu.eg; 4 Faculty of Nanotechnology for Postgraduate Studies, Sheikh Zayed Branch Campus, Cairo University, Sheikh Zayed, Giza 12588, Egypt; drsameheltayer@yahoo.com; 5 Department of Biochemistry, Faculty of Pharmacy, Cairo University, Kasr El-Aini Street, Cairo 11562, Egypt; nadine.hanna@pharma.cu.edu.eg (N.W.M.); mai.azzam@pharma.cu.edu.eg (M.A.A.); 6 Max Planck Institute of Molecular Plant Physiology, Am Mühlenberg 1, 14476 Potsdam-Golm, Germany; alseekh@mpimp-golm.mpg.de (S.A.); fernie@mpimp-golm.mpg.de (A.R.F.); 7 Center for Plant Systems Biology and Biotechnology, 4000 Plovdiv, Bulgaria; 8 Pharmacognosy Department, Faculty of Pharmacy, Cairo University, Kasr El-Ainy Street, Cairo 11562, Egypt; maha.salama@pharma.cu.edu.eg; 9 Department of Pharmacognosy, Faculty of Pharmacy, The British University in Egypt, Suez Desert Road, El Sherouk City, Cairo 11837, Egypt; 10 Department of Pharmacognosy, Faculty of Pharmacy, October University for Modern Sciences and Arts (MSA), Giza 12451, Egypt

**Keywords:** obesity, metabolomics, green tea, white tea, oolong tea, catechins

## Abstract

White, green, and oolong teas are produced from the tea plant (*Camellia sinensis* (L.) Kuntze) and are reported to have anti-obesity and hypolipidemic effects. The current study aims to investigate the anti-obesity effects of a tea mixture nano-formulation by targeting the AMPK/Sirt-1/GLUT-4 axis in rats. In vitro lipase and *α*-amylase inhibition assays were used to determine the active sample, which was then incorporated into a nanoparticle formulation subjected to in vivo anti-obesity testing in rats by measuring the expression level of different genes implicated in adipogenesis and inflammation using qRT-PCR. Moreover, metabolomic analysis was performed for each tea extract using LC/ESI MS/MS coupled to chemometrics in an attempt to find a correlation between the constituents of the extracts and their biological activity. The in vitro pancreatic lipase and *α*-amylase inhibition assays demonstrated more effective activity in the tea mixture than the standards, orlistat and acarbose, respectively, and each tea alone. Thus, the herbal tea mixture and its nanoparticle formulation were evaluated for their in vivo anti-obesity activity. Intriguingly, the tea mixture significantly decreased the serum levels of glucose and triglycerides and increased the mRNA expression of GLUT-4, P-AMPK, Sirt-1, and PPAR-γ, which induce lipolysis while also decreasing the mRNA expression of TNF-α and ADD1/SREBP-1c, thereby inhibiting the inflammation associated with obesity. Our study suggests that the tea mixture nano-formulation is a promising therapeutic agent in the treatment of obesity and may also be beneficial in other metabolic disorders by targeting the AMPK/Sirt-1/Glut-4 pathway.

## 1. Introduction

One of the most common health problems worldwide is obesity; according to the WHO, it is defined as the excessive or abnormal accumulation of fats. It is not just a cosmetic concern, but also a medical condition occurring among diverse age groups that increases the risk of health problems and leads to numerous complications [1]. Obesity has been increasing in prevalence worldwide to the extent that it is currently considered an epidemic condition [2]. According to a recent study that covered obesity prevalence over one hundred and ninety-five countries, Egypt had the highest rates of adult obesity while the USA scored the highest rates of childhood obesity [3].

Obesity is an imbalance between energy expenditure and caloric intake. This imbalance results from the interactions between several factors that include both environmental and genetic factors; hence, obesity is known to be a multifactorial complex disorder. Many controversial studies have indicated the consequences of gene mutation in increasing BMI. These include genes involved in the regulation of food intake or metabolism such as peroxisome proliferator-activated receptor gamma (PPAR-γ), the melanocortin-4 receptor (MC4R) gene, the proopiomelanocortin (POMC) gene, the leptin gene, and the fatty acid synthase (FAS) gene [4]. Genetics aside, other risk factors can lead to the development of obesity, such as socioeconomic factors; physical inactivity; unhealthy lifestyle; stress; drugs, including antidiabetic drugs, antidepressants, and steroids; and diseases like Cushing syndrome and metabolic syndrome [5]. When one becomes obese, they become more susceptible to developing health problems and life-threatening diseases such as hypertension, diabetes, coronary heart disease, dyslipidemia, and cancer [6].

The main approach for reducing weight is through exercise and eating a balanced, healthy diet; however, for some obese people, this approach is not effective or not enough. Thus, they tend to take weight-reducing drugs alongside changing their lifestyle. One of the most common medications used is orlistat, commonly known and marketed as Orly or Xenical^®^, which reduces weight by decreasing fat absorption by inhibiting gastric and pancreatic lipases and positively affecting blood pressure, insulin resistance, and lipid levels. Lorcaserin is another drug that reduces the appetite by stimulating the serotonin 2C receptor, which suppresses the appetite [5]. Nonetheless, these medications have many side effects, such as gastrointestinal tract disturbances, caused by orlistat intake [7], or an increase in heart rate, caused by the administration of phentermine–topiramate [8]. Given these side effects, trends in treating obesity have been inclining toward traditional herbal medicine, which has been used in therapy, proving it can be a promising approach.

Natural products have been proposed to counteract obesity by acting on multiple molecular targets [9]. For example, ginger stimulates lipolysis, inhibits adipogenesis by blocking PPAR-γ action, and prevents lipid droplet accumulation in 3T3-L1 pre-adipocytes [10]. Additionally, *Moringa oleifera* has proven efficacy in reducing weight by up-regulating the mRNA expression of adiponectin, which is the main pathway for adiposity enhancement, and downregulating the mRNA expression of resistin and leptin, which, in turn, reduces insulin resistance [11].

Tea, whether green, oolong, or white, is commonly used for its health benefits and is traditionally reported to have anti-obesity and hypolipidemic effects [12,13,14]. Several studies have suggested its mechanisms in weight-reducing action but not in combination. Oolong tea contains abundant bioactive constituents such as caffeine and polyphenols that make it a powerful remedy for accelerating lipolysis and inhibiting dietary fat absorption [1]. Also, green tea, with its main constituents, catechins, possesses anti-obesity action by increasing energy expenditure and fat oxidation and downregulating hepatic enzymes that are involved in lipid metabolism [15] It also upregulates adiponectin levels by inhibiting ERK; increases the phosphorylation of PPAR-γ; and, consequently, increases its expression [16].

Moreover, white tea has been investigated by several studies and has been shown to exhibit an anti-obesity effect through its lipolytic activity and a reduction in triglycerides via its active constituents, methylxanthines [13]. Also, white tea inhibits adipogenesis with a decrease in adipogenesis-linked transcription factors, which include PPARγ, ADD1/SREBP-1c (alpha-adducin/sterol regulatory element-binding protein-1c), and an increase in the gene expression of Sirt-1, which, in turn, increases insulin sensitivity and reduces adipogenesis [17].

Moreover, the use of nanoparticles in the treatment of obesity could lead to a better and improved weight management system because of its better dissolution, absorption, and bioavailability; its ability to safely and effectively target tissues or organs of interest; and decreased therapeutic range fluctuation. However, nanoparticles have several disadvantages, such as their short biological half-life, expenses for labor, intensity, and high tendency to aggregate [18].

The primary cellular energy sensor is AMP-activated protein kinase (AMPK), a ubiquitous serine/threonine protein kinase. To regain energy equilibrium, activated AMPK enhances catabolic and inhibits anabolic metabolism. Furthermore, in metabolically sensitive tissues (such as the hepatic, adipose, and skeletal muscles), active AMPK improves the performance of NAD-dependent deacetylase sirtuin-1 (Sirt-1), a mechanism associated with aging that influences the accumulation of fat [19].

Increasing evidence indicates Sirt-1 has a potential role in controlling lipid and glucose metabolism. Additionally, Sirt-1 overexpression protects against metabolic abnormalities via high-fat diets and insulin resistance in diabetic animals. Sirt-1 controls several transcription factors in endocrine signaling pathways, most notably glucose transporter-4 (GLUT-4), therefore improving insulin sensitivity [20].

The aim of this study was, thus, to examine the in vitro lipase inhibition and *α*-amylase inhibition activities of each tea extract (green tea, white tea, and oolong tea) and a mixture of the three teas. Moreover, we analyzed the metabolic profile of each tea extract in an attempt to identify correlations between the constituents of the extracts and their observed biological activity. We additionally investigated the in vivo effect of a nano-sized herbal tea mixture on obesity induced by a high-fat diet in rats in an attempt to provide insight into their molecular mode of action by targeting the AMPK/Sirt-1/GLUT-4 axis.

## 2. Materials and Methods

### 2.1. General

Folin–Ciocalteu reagent, sodium carbonate, rutin standard, sodium nitrite, aluminum trichloride, methanol, gallic acid, orlistat, type II crude porcine pancreatic lipase, 4-nitrophenyl octanoate (NPC), dimethylsulfoxide, Tris-HCl buffer (pH = 8.5), acarbose, potato starch, sodium phosphate buffer, sodium chloride, *α*-amylase, sodium potassium tartrate tetrahydrate, sodium hydroxide, and 3,5-dinitro salicylic acid were all purchased from Sigma Aldrich, Munich, Germany. A FluoStar Omega microplate reader (Shimadzu UV-1650PC, Kyoto, Japan) was used for the determination of total phenolics and flavonoid content. A plate reader (Model: BioTek ELX 808, Milano, Italy) was also used for a pancreatic lipase inhibition test. MaoFeng green tea (cultivated in China) and Bai Mu Dan white tea (cultivated in China) were purchased from the United Arab Emirates, while Tieguanyin Oolong tea (cultivated in Anxi Fujian in China) was purchased from Malaysia. Ethanol (95% (*v*/*v*)) was obtained from Sigma Aldrich (Germany).

### 2.2. Plant Material and Extraction

The powders of each tea (400 g) were extracted via cold maceration at 4 °C using 1:1 ethanol (95%): water (4 times × 2 L). For each extraction, flasks were shaken at 4 °C for 30 min and filtered, and the marc was subjected to subsequent extraction.

Finally, evaporation of the pooled extracts occurred under reduced pressure using a rotary evaporator at a temperature not exceeding 60 °C to yield 109, 56, and 70 g of white, green, and oolong tea, respectively.

### 2.3. UPLC–HRMS-MS Analysis

An analytical method consisting of ultrahigh-performance liquid chromatography coupled with high-resolution tandem mass spectrometry (UPLC–HRMS-MS) was applied [21]. The UPLC system consisted of a Waters Acquity UPLC system (Waters, Machester, UK) connected to an Exactive Orbitrap tandem mass spectrometer (Thermo-Fisher, Bremen, Germany) via an electrospray ionization interface. Furthermore, an RP High-Strength Silica (HSS) T3 column (100 mm × 2.1 mm, containing 1.8 μm diameter particles C18) was offered by Waters, Manchester, UK. Samples (4 µL, each) were injected at a flow rate of 0.4 mL min^−1^, and the column temperature was set to 30 °C. The mobile phase consisted of 0.1% formic acid in water (phase A) and 0.1% formic acid in methanol (phase B), using a gradient elution of 1% B for 0–1 min; 1–40% B for 1–11 min; 40–70% B for 11–13 min; 70–99% B for 13–15 min; 99–1% B for 16–17 min; and 1% B for 17–20 min. The mass spectrometer was operated in positive (+ESI) mode, and the spectra were recorded alternating between full-scan and all-ion fragmentation scan modes, covering a mass range from 100 to 1500 *m*/*z*. The parameters for analysis were performed as follows: spray voltage, 3.5 kV; capillary temperature, 150 °C; auxiliary gas heating temperature, 300 °C; sheath gas, 60 units; auxiliary gas, 35 units; skimmer voltage, 25 V; and tube lens, 130 V [21]. Data processing was achieved using the ToxID 2.1.2 and Xcalibur 2.1 software package (Thermo Fisher Scientific Inc., Waltham, MA, USA). Multivariate analysis was performed using MetaboAnalyst 5.0 [22].

### 2.4. Determination of Total Phenolics

Standards for total phenolics were prepared by dissolving gallic acid (1 mg/mL) in methanol, and seven serial dilutions of 500, 250, 125, 62.5, 31.2, 15.6, and 7.8 μg/mL were prepared. Sample solutions of white, green, and oolong teas were prepared at a concentration of 1 mg/mL in methanol. The Folin–Ciocalteu colorimetric method was used in the spectrophotometric determination of the total phenolic content of the three extracts as the gallic acid equivalents [23].

### 2.5. Determination of Total Flavonoids

Standards for total flavonoids were prepared by dissolving rutin at a concentration of 1 mg/mL in methanol. Then, six serial dilutions at concentrations of 1000, 500, 250, 150, 100, and 50 μg/mL were prepared. Sample solutions of white, green, and oolong teas were prepared in a concentration of 0.5 mg/mL in methanol. The total flavonoid contents were determined using the aluminum chloride colorimetric method, following Park et al.’s method [24].

### 2.6. Pancreatic Lipase Inhibition

The method of Slanc et al. was used for the determination of the lipase inhibition capacity of the tested extracts using 4-nitrophenyl octanoate as a substrate and compared to the positive control, orlistat [25]. The test was performed for each extract and then for a mixture of the three extracts (1:1:1 *w*/*w*/*w*). The ELX 808 was used to measure the absorbance at 412 nm. The percentage of inhibition was calculated using the following equation: 100 − [(sample/control) × 100].

### 2.7. Alpha-Amylase Inhibition

Potato starch was stirred in 6.7 mmol/L sodium chloride with 20 mmol/L sodium phosphate buffer to obtain a starch solution (0.5% *w*/*v*), pH 6.9 at 20 °C. *α*-Amylase (25.3 mg, 10 U/mg) was mixed with 100 mL of cold distilled water for the preparation of the enzyme solution. The extract was dissolved in a buffer to obtain a concentration ranging from 1000 µg/mL to 31.25 µg/mL. A sodium potassium tartrate solution (12.0 g of sodium potassium tartrate tetrahydrate in 8.0 mL of 2 M NaOH) was mixed with a 96 mmol/L 3,5-dinitro salicylic acid solution and used as an indicator. The positive control was acarbose. The test was performed on each extract and then on a mixture of the three tea extracts (1:1:1: *w*/*w*/*w*). The ELX 808 was used to measure the absorbance at 540 nm. The percentage of inhibition was calculated using the following equation: 100 − [(sample/control) × 100] [26].

### 2.8. Synthesis of Tea Extract Nanoparticles

Synthesis was carried out with a sonochemical method using an ultrasonic prop instrument (Hielscher model, up400s; Teltow, Germany) with polymeric stabilization. In brief, 20 g of tea extract was added to 1 L of doubled deionized water and subjected to ultrasonic pressure under the following conditions: increases every 2 s at 95% amplitude power and maximum temperature (60 °C); rest time, 1 sec.; and total time of sonication, 4 h. Ultrasonic irradiation led to the formation and growth of micron-sized bubbles, which had extreme temperatures and pressure on their inner and outer sides. When collapsing, the tea molecules precipitated via nucleated formation; rapid cooling followed, leading to the synthesis of tea nanoparticles. Polyvinyl alcohol (PVA, 0.01 g) was added to the mixture, which was resubjected to ultrasonic pressure under the following conditions: increases every 2 s at 50% amplitude power and maximum temperature (60 °C); rest time, 1 s.; and the total time of sonication was one hour.

### 2.9. Nanoparticle Characterization

Characterization was restricted to the identification of the shape and size of tea nanoparticles coated with PVA. Shape identification was carried out with an atomic force microscope (AFM) and a high-resolution transmission electron microscope (HRTEM). Their shapes and textures were scanned using a high-resolution transmission electron microscope with an accelerating voltage of 250 kV and magnification of 20×. Samples were prepared before measurement via sonicated irradiation using a sonication prop under the following conditions: increases every 3 s at 73% amplitude power and maximum temperature for 33 minutes. Finally, 40 microns were added to the TEM grade via air drying for 4.5 h [27]. The AFM had a study area of 300 nm × 300 nm and used an Al tip in contact mode and vacuum conditions. Thin film formation needed to be performed first via the free precipitation of the samples on mica plates [28]. The size was measured using dynamic light scattering (DLS). In DLS analysis, the sample suspension is illuminated by a laser beam, after which, the laser light scatters in all directions. The light scattering is observed at a certain angle over time. Signal variation is due to the random Brownian motion of the particles. Angular intensity distribution is used to determine the particle size via the Stokes–Einstein equation [29].

### 2.10. The Experimental In Vivo Study

#### 2.10.1. Animals

Forty Wister albino male rats, 100–120 g were purchased from a local supplier. The rats were housed in the MSA University animal house in standard environmental conditions: humidity, 40–60%; temperature, 22 ± 2° C; and a 12 h dark/light cycle. All the animal experiments were approved by the MSA Institutional Ethical Committee under number (BP3/Ec3/2022PD). Rats were given one week to adapt to their new environment and were given normal food that week.

#### 2.10.2. High-Fat Diet Induction

Control rats were fed a typical laboratory rodent diet. To demonstrate diet-induced obesity, a high-fat diet (HFD) was given to the remaining rats for three months. The formulation and assembly of the HFD were formerly explained in [30]. Throughout the entire investigation, the rats’ weights were recorded weekly using a digital balance scale.

#### 2.10.3. Experimental Design

The rats were divided into eight groups: the control group (normal diet), the high-fat-diet (HFD) group, the stop HFD group, the low-dose (100 mg/kg) conventional-tea-treated group, the low-dose (100 mg/kg) nano-formulated-tea-treated group, the high-dose (300 mg/kg) conventional-tea-treated group, the high-dose (300 mg/kg) nano-formulated-tea-treated group, and the orlistat (200 mg/kg) group. Every five rats were separated into well-closed cages that had access to food and water.

All rats were weighed before starting HFD. Then, the rats were fed daily with the HFD for three months before starting the treatment, except the control group, which continued on a normal diet. After three months, all groups stopped the HFD, except the HFD group, and continued on a normal diet, and treatment started. The treated groups were orally intubated via intragastric gavage using an exact dose based on the rats’ weights for 5 weeks.

#### 2.10.4. Sample Collection and Biochemical Analysis

At the end of the study, the final body weight of the rats was recorded; then, all rats were sacrificed via cervical dislocation under ether anesthesia. Blood samples were collected after fasting the rats overnight in commercially available serum-collecting tubes, centrifuged at 4000× *g* for 10 min at room temperature, and stored at −80 °C for the analysis of biochemical parameters; serum glucose, creatinine, aspartate transaminase, and alanine transaminase were measured enzymatically (Spinreact, Girona, Spain; ref: 41010, ref: 1001110, ref: 41270, ref: 41280, respectively), and the lipid profile (total cholesterol (TC), high-density lipoprotein cholesterol (HDL-C), low-density lipoprotein cholesterol (LDL-C), and triglycerides (TGs)) was analyzed enzymatically (Spinreact, Spain; ref: 41020, ref: 1001095, ref: 41023, ref: 41030, respectively).

#### 2.10.5. Adipose Tissue Collection and Analysis

White adipose tissue was collected from the epididymal, mesenteric, and perirenal area, rinsed twice with saline, and weighed immediately; then, the adipose tissue index was calculated according to the following formula: (API = retroperitoneal adipose tissue ×100).

Part of the adipose tissue was ground using a tissue homogenizer (Stuart Homogeniser SHM1 & SHM3, STU0005/Version 1.0, Cadmus, Chelmsford, UK) and then mixed in 650–800 µL of sterile, ice–cold, pH 7.3, phosphate-buffered saline (PBS). The incubated mixture was put on ice, subjected to 20–30 s of sonication, and then centrifuged for 15 min at 3000× *g*. The supernatant was collected for the measurement of leptin (CUSABIO, Houston, TX, USA, ref: CSB-E07433r), adiponectin (RayBiotech, Inc., Peachtree Corners, GA, USA), vaspin (MyBioSource, Inc., San Diego, CA, USA, ref: MBS2501454), and omentin (MyBioSource, ref: MBS701307) using a rat enzyme-linked immunosorbent assay (ELISA) kit according to the manufacturer’s instructions.

#### 2.10.6. Gene Expression and qRT-PCR

Tumor necrosis factor-alpha (TNF-*α*), glucose transporter type-4 (GLUT-4), sirtuin-1 (Sirt-1), peroxisome proliferator-activated receptor gamma (PPAR-γ), and phosphorylated (activated) protein kinase (P-AMPK) RNA were extracted from the adipose tissue, and differentiation-dependent factor 1 (ADD1/SREBP-1c) was extracted from the livers of all rats in the different groups using the RNeasy Mini-Kit (QIAGEN, Hilden, Germany, ref: 74106) according to the manufacturer’s instructions. The extracted RNA was eluted in 30 μL of nuclease-free distilled water. The total RNA of each sample was used for cDNA conversion using a high-capacity cDNA reverse transcription kit (Fermentas, Waltham, MA, USA). The quantification of TNF-*α*, GLUT-4, sirt-1, PPAR-γ, P-AMPK, and ADD1/SREBP-1c was determined using the SYBR^®^ Green Quantitative RT-qPCR Kit (Sigma, Darmstadt, Germany ref: QR0100) in the StepOnePlus™ Real-Time PCR System (Thermofisher, Waltham, MA, USA, ref: 4376600) with primers purchased from QIAGEN and a PCR array according to manufacturer’s instructions.

## 3. Results

### 3.1. Identification of Metabolites in the Tea Samples Following UPLC–HRMS-MS Analysis

**Figure 1:**
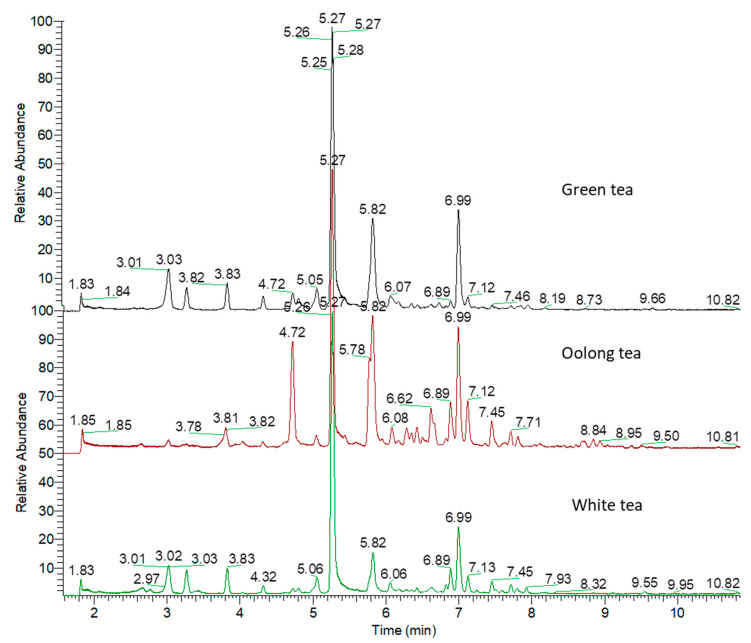
Overlaid chromatograms of tea samples as analyzed using ultrahigh-performance liquid chromatography–tandem mass spectrometry (UPLC-MS/MS).

An analytical method consisting of liquid chromatography coupled with high-resolution tandem mass spectrometry (LC–HRMS-MS) was performed for the analysis of metabolites from three types of tea (green, white, and oolong tea; Figure 1). The identification of the compounds was based on their retention times and mass spectra in full-scan mode (MS) and all-ion fragmentation (AIF) MS/MS mode. In total, we annotated 49 compounds in three kinds of tea samples, which are provided in Table S1.

**Figure 2:**
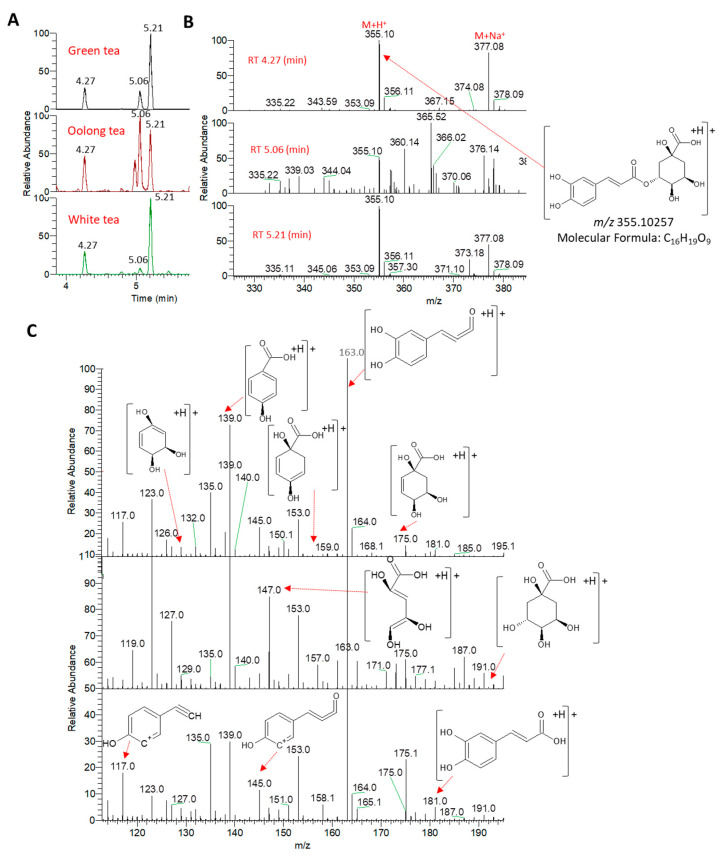
UPLC-MS analysis of caffeoylquinic acid isomers. (**A**) Elution pattern of three caffeoylquinic acid isomers for the peak extracted at 355.10257 *m*/*z*. (**B**) Positive electrospray ionization full-scan mass spectrometry (ESI-MS) analysis (**C**). Positive electrospray ionization–tandem mass spectrometry (ESI-MS/MS) analysis.

Chlorogenic acids, which are composed of quinic acid linked via an ester bond to caffeic acid, are considered the most abundant phenolics in the human diet. The three most common mono-caffeoylquinic acids—namely, 3-*O*-caffeoylquinic acid (3-CQA A; neochlorogenic acid), 4-*O*-caffeoylquinic acid (4-CQA; cryptochlorogenic acid), and 5-*O*-caffeoylquinic acid (5-CQA; A; chlorogenic acid)—were annotated from tested tea samples using positive ionization mode. Chlorogenic acid isomers were thoroughly ionized and identified in negative ionization mode, owing to the presence of a carboxylic acid functional group [31,32]. Despite being structural isomers with the same protonated ion at 355.10257 *m*/*z*, the elution pattern and MS/MS data allowed for the annotation of the three isomers. We and others have shown previously that the elution pattern of these isomers can be denoted as 3-CQA < 5-CQA < 4-CQA [32,33]. Consistently, the extracted ion chromatograms of the peak at 355.10257 *m*/*z* demonstrated three compounds with the same elution pattern described before (Figure 2).

From the MS/MS spectra, the fragmentation pattern of the protonated caffeoylquinic acid provided the most characteristic ions at 181.04941 *m*/*z* and 193.04814 *m*/*z*, representing protonated caffeic and quinic acids, respectively (Figure 2). The loss of one or two water molecule(s) from the protonated caffeic acid provided characteristic fragments at 163.03885 and 145.02814 *m*/*z*, respectively. The decarbonylation of the ion at 145.02814 *m*/*z* provided a fragment at 117.03364 *m*/*z* (Figure 2 and Figure S1). Additionally, the loss of one water molecule from the protonated quinic acid provided a characteristic ion at 175.10738 *m*/*z*. The loss of one or two water molecule(s) from this ion provided the characteristic fragments at 157.02797 and 139.03856 *m*/*z*, respectively. Dehydrated quinic acid can be further fragmented to provide ions at 147.04356 *m*/*z* (quinic acid+H-H_2_O-CH_2_=CH_2_)^+^ and 129.03323 *m*/*z* (quinic acid+H-H_2_O-HCOOH)^+^ [31]. Intriguingly, the fragmentation pattern of the 5-CQA isomer showed a characteristic and base peak ion for the dehydration of the protonated quinic acid at 163.03885 *m*/*z* (Figure S2). Additionally, 3-CQA showed a characteristic and base peak ion at 139.03856 *m*/*z* for the loss of three water molecules from the protonated quinic acid (Figure S2). Further, the base peak fragment ion at 163.03885 *m*/*z*, as well as the characteristic fragment at 117.03364 *m*/*z*, marked 4-COA.

The main observed flavonoid glycosides were flavonol derivatives, mainly kaempferol, quercetin, and myricetin. The peaks at retention times of 7.44 and 7.71 min presented ions at 595.1664 and 449.1085 *m*/*z,* corresponding to protonated adducts (M+H)^+^ (Figure S3). These ions released characteristic and base peak MS^2^ fragments at 287.05 *m*/*z*. These fragment ions correspond to the loss of a hexosyl moiety (e.g., glucose, −162 u) and a deoxyhexosyl–hexoside moiety (e.g., glucose and rhamnose, −308 u), providing a kaempferol moiety. The fragment ion corresponds to the loss of a deoxyhexosyl moiety (−146 u), which was observed at 449.1085 *m*/*z*. The mass characteristics of the peak at 449.1085 *m*/*z* indicated that it corresponds to a kaempferol derivative bearing a hexosyl moiety. Nevertheless, fragment ions for the peak at 595.1664 were detected at 449.11 and 433.11 *m*/*z*, which correspond to the loss of a deoxyhexosyl moiety (−146 u) and a hexosyl moiety (−162 u), respectively. This observation of alternative losses points out the possible different locations of sugar moieties on the aglycone. Therefore, these peaks were assigned as kaempferol glucoside–rhamnoside and kaempferol glucoside, which have been identified before from green tea samples [34,35].

Catechin, gallocatechin, and gallocatechin 3-*O*-gallate are known as major tea catechins, together with their *epi*-isomers. A peak was detected at RT 6.08 min showing a protonated molecule (M+H)^+^ at 459.0927 *m*/*z*. MS^2^ fragment ions were detected at 307.08 *m*/*z* [M+H-152]^+^ because of the loss of a galloyl moiety (−152 u) and at 289.07 *m*/*z* because of the loss of a water molecule from a fragment ion at 307.08 (Figure S4). The characteristic fragmentation pattern allowed us to annotate catechin, gallocatechin, and gallocatechin 3-*O*-gallate at 5.77, 4.72, and 6.08 min, showing fragment ions at 291.0862 (mass error, −0.35 ppm), 307.0813 (0.13 ppm), and 459.0927 (1.01 ppm) *m*/*z*, corresponding to the protonated adducts (M+H)^+^.

### 3.2. Multivariate Analysis of Metabolic Differentiation of Green, White, and Oolong Tea Samples

**Figure 3:**
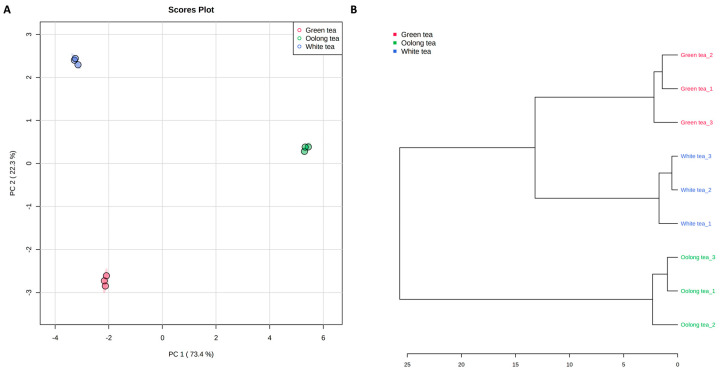
Principal component analysis (**A**) and hierarchical cluster analysis (**B**).

The relative abundances of the annotated metabolites were variable based on the tea type. Using unsupervised pattern recognition methods such as principal component analysis (PCA) and hierarchical cluster analysis (HCA), the analyzed samples were distinguished, indicating significant differences in their metabolomes (Figure 3).

**Figure 4:**
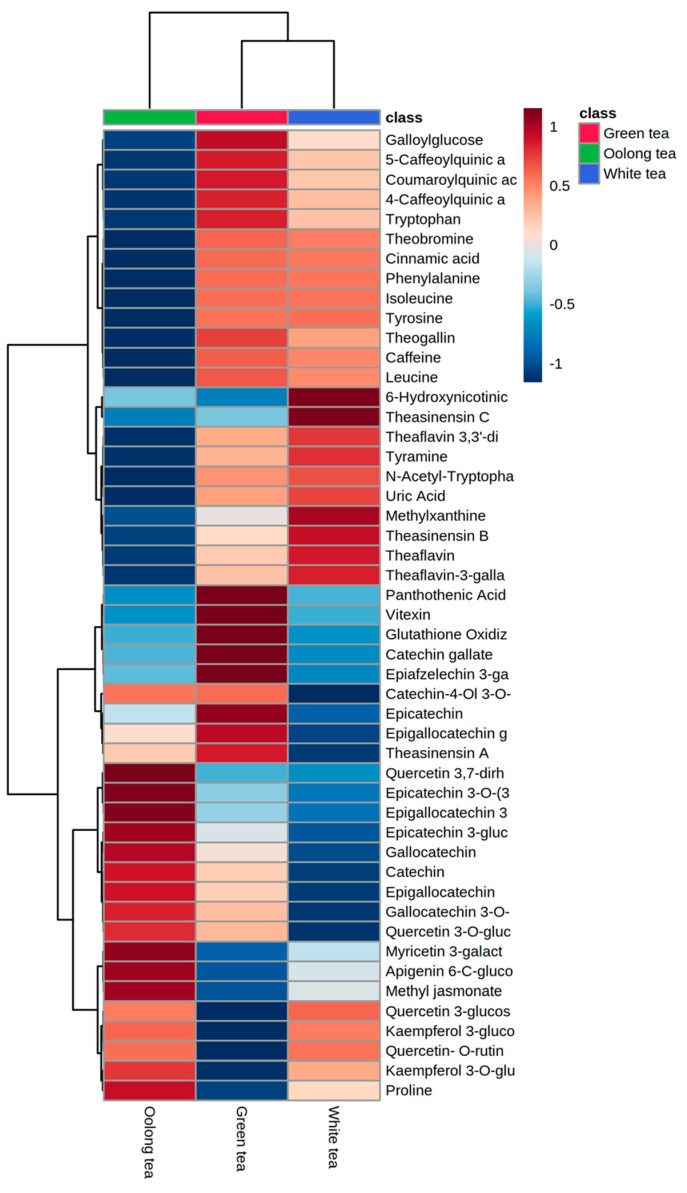
Heatmap showing a visualization of clustering analyses of metabolites identified from the different tea samples. Each row represents a metabolite, and each column represents the average of samples from each type (*n* = 3). Red and blue denote high and low relative abundances of metabolites transformed into the log_10_ scale, respectively.

Green and white tea samples were clustered from oolong tea samples. Oolong and green tea samples showed a higher abundance of catechins, such as catechin, epicatechin, epigallocatechin, epigallocatechin gallate, gallocatechin, and gallocatechin 3-*O*-gallate, than white tea (Figure 4). These results are also in good agreement with previous studies [36]. Green and white tea samples showed a higher abundance of metabolites from alkaloid, amino acid, organic acid, and phenolic acid chemical classes than oolong tea. The abundances of most of the detected amino acids, such as isoleucine, leucine, phenylalanine, tryptophan, tyramine, and tyrosine, were in the order green tea > white tea > oolong tea. Dimeric catechins such as theaflavin, theaflavin 3,3′-gallate, theaflavin-3-gallate, theasinensin B, and theasinensin C dominated the white tea samples.

### 3.3. Total Phenolics and Total Flavonoids

The total phenolics were found to be 474.3, 338.4, and 294.25 mg GAE/g extract in green, white, and oolong tea, respectively. On the other hand, the total flavonoids were found to be 325.3, 273.2, and 61.3 mg rutin equivalent/g extract, respectively.

### 3.4. Pancreatic Lipase Inhibition

The results showed that the three extracts had percentage inhibitions at 100 µg/mL of 87.9, 83.7, and 85.13% for green, white, and oolong tea, respectively. Meanwhile, the mixture showed a percentage inhibition of 86.09% for 100 µg/mL, while orlistat had a percentage inhibition of 70.1%. Concerning IC_50_ values, green tea had an IC_50_ of 14.25 ± 5.4 µg/mL, white tea showed an IC_50_ of 16.5 ± 4.5 µg/mL, oolong tea had an IC_50_ of 15.45 ± 4.2 µg/mL, the mixture had an IC_50_ of 12.2 ± 3.9 µg/mL, and orlistat had an IC_50_ of 28.96 ± 6.4 µg/mL.

### 3.5. α-Amylase Inhibition

At a concentration of 600 µg/mL, green tea had a percentage inhibition of 72.2 ± 6.7%, white tea had a percentage inhibition of 71.5 ± 7.8% for 600 µg/mL, and oolong tea had a percentage inhibition of 65.4 ± 7.5%. Meanwhile, the mixture had a percentage inhibition of 73.4 ± 8.6% for 600 µg/mL, and acarbose had a percentage inhibition of 70.1 ± 7.5%. IC_50_ is the concentration necessary to inhibit 50% of the enzyme concentration. The IC_50_ for green tea was 181.79 ± 8.5 µg/mL, that of white tea was 192.07 ± 7.75 µg/mL, oolong tea was 256.18 µg/mL, that of the mixture was 176.19 ± 8.9 µg/mL, and acarbose had an IC_50_ of 225 ± 5.9 µg/mL. The top metabolites correlating with anti-obesity activity represented by pancreatic lipase inhibition activity were picked by analyzing Pearson’s correlation coefficients (Figure S5).

### 3.6. Shape Characterization

#### 3.6.1. High-Resolution Transmission Electron Microscope (HRTEM) Observation

**Figure 5:**
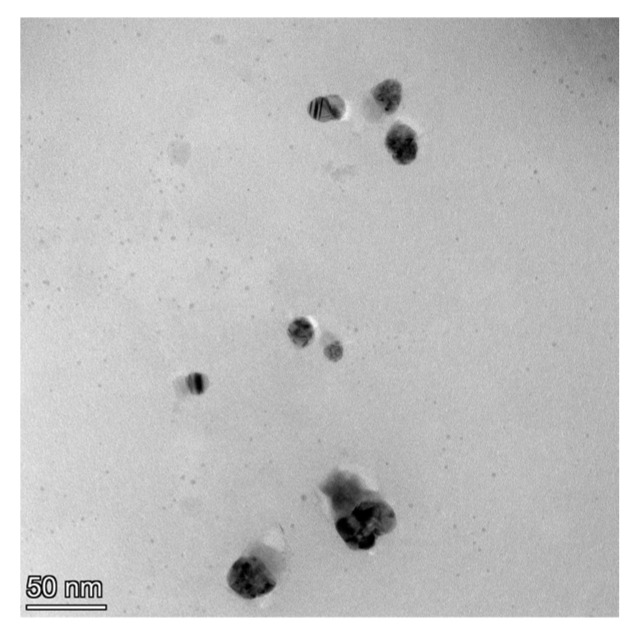
TEM images of tea nanoparticles coated with PVA.

Figure 5 shows TEM images of tea nanoparticles coated with PVA: spherical tea nanoparticles (dark color) with homogenous shape and size distributions were coated with a thin PVA layer (light color). The sizes in the TEM image range between 50 and 65 nm. The TEM instrument was manufactured by the FEI Tecnai company, model G2 F20.

#### 3.6.2. Atomic Force Microscope (AFM)

**Figure 6:**
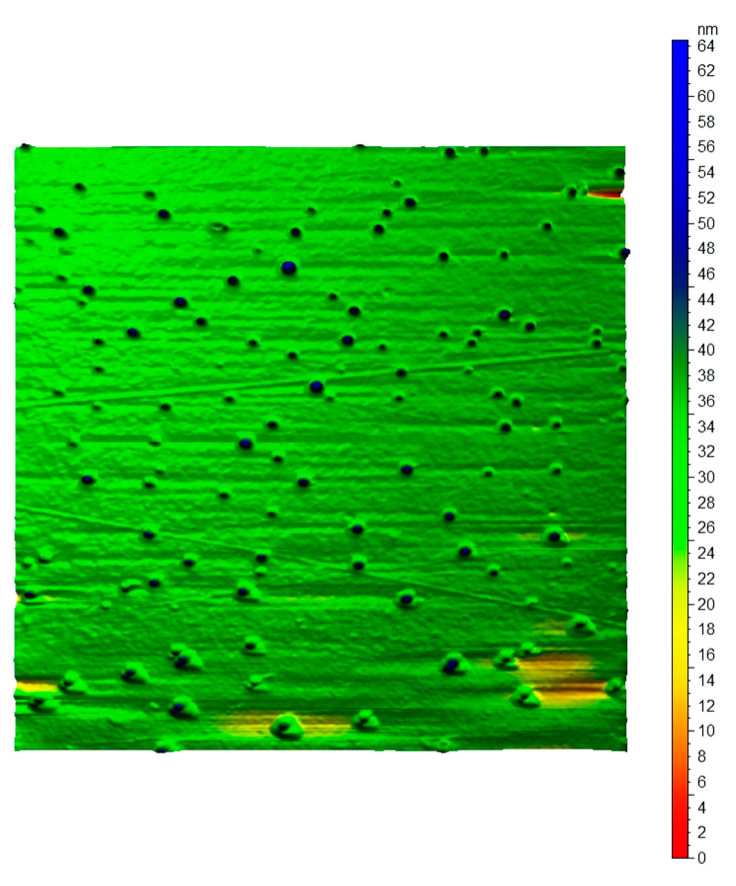
Top view AFM image of tea nanoparticles coated with PVA.

**Figure 7:**
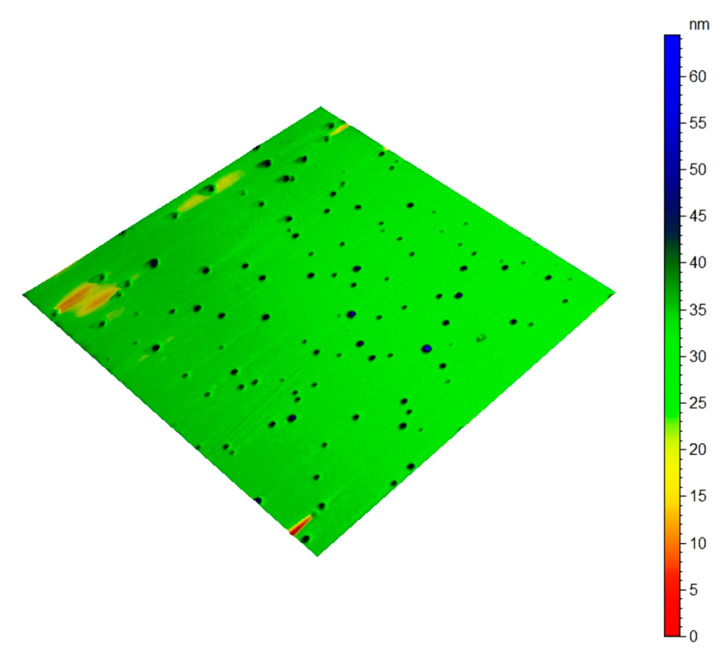
Three-dimensional AFM image of tea nanoparticles coated with PVA.

AFM images of tea nanoparticles coated with PVA, as shown in Figure 6 and Figure 7, conformed with the shapes, sizes, concentrations, and agglomerations obtained from the TEM images. The AFM images illustrate the spherical shape of the tea nanoparticles (blue color) fully surrounded by a PVA layer (green color). The AFM image size is 1µm x1µm, with a maximum tea nanoparticle height ranging between 45 and 65 nm. The AFM images were created using an AFM instrument manufactured by Agilent Technology (Stevens, Creek Blvd, Santa Clara, CA, USA), model 5600LS.

#### 3.6.3. Size Characterization

##### Dynamic Light Scattering (DLS)

**Figure 8:**
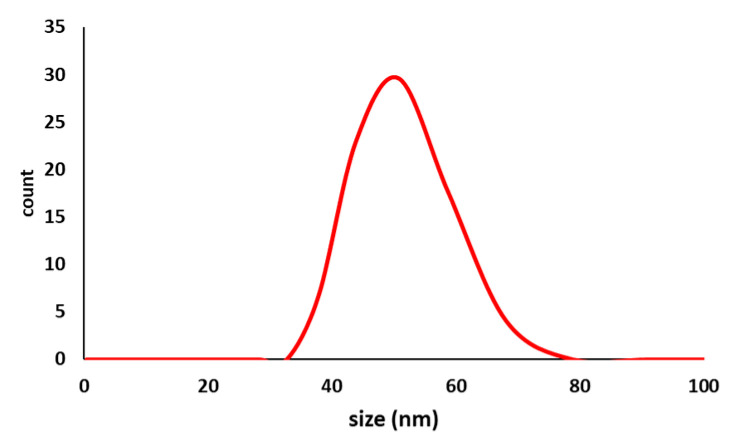
Three-dimensional DLS chart of tea nanoparticles coated with PVA.

Figure 8 shows the DLS of tea nanoparticles coated with PVA. The DLS results agree with the size obtained from the AFM and TEM images. However, the size obtained from DLS was 55 nm. with a homogenous size distribution. DLS was achieved using the Nano Sight NS500, manufactured by Malvern Panalytical Ltd., Engima Business Park, Grovewood Road, Malvern, UK.

### 3.7. Effect of Conventional and Nano-Tea Mixture Extracts on Wister Rats’ Weight and Adipose Tissue Deposition

**Figure 9:**
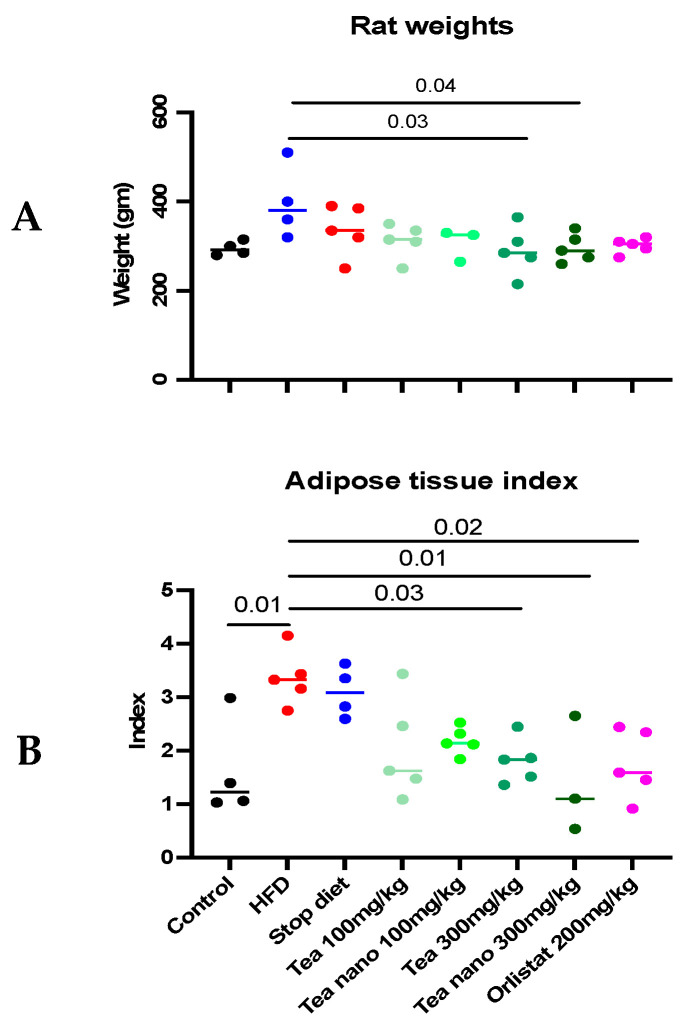
Effects of conventional and nano-tea mixture extracts on (**A**) Wister rat weight and (**B**) adipose tissue deposition. Data represented as scatter plots; significant at *p* < 0.05.

After five weeks of daily treatment with two different doses of conventional and nano-formulated herbal tea mixture extracts, there was a statistically significant difference between the HFD group and the high-dose group (300 mg/kg) in the conventional and nano-formulated groups, as seen in Figure 9A. The weights of the 300 mg tea group and 300 mg nano-tea group were significantly decreased when compared with the HFD group (*p* = 0.0309 and *p* = 0.0483, respectively). On the other hand, there was no significant difference when the other groups were compared to the HFD group.

Fat deposition in the rats’ adipose tissues showed a statistically significant increase in the HFD group when compared with the control groups (*p* = 0.01). This showed a statistically significant decrease in the high-dose groups (300 mg/kg) in the conventional and nano-formulated groups, as well as the orlistat group, when compared with the HFD group (*p* = 0.03, 0.01, and 0.02, respectively), and no differences compared to the low-dose (100 mg/kg) groups, as shown in Figure 9B.

### 3.8. Effect of Herbal Treatment on Lipid Profile, Blood Glucose Levels, and Kidney and Liver Functions

**Figure 10:**
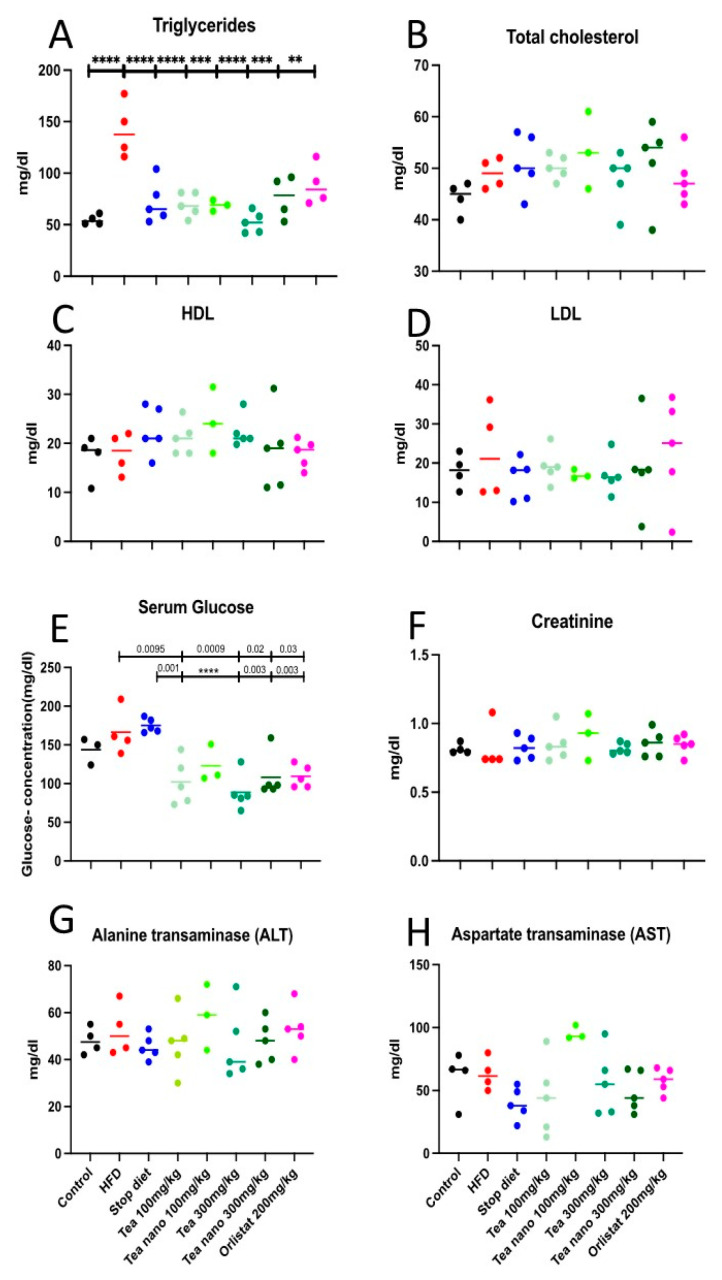
Effects of conventional and nano-tea mixture extracts on lipid profiles, (**A**) Triglyceride, (**B**) Total cholesterol, (**C**) HDL, (**D**) LDL, and (**E**) blood glucose levels, as well as kidney and liver function represented as (**F**) Creatinine, (**G**) ALT and (**H**) AST. Data represented as scatter plots; significant at *p* < 0.05 are represented as ** (*p* < 0.01), *** (*p* < 0.001) and **** (*p* < 0.0001). Different colors are indicating different groups as shown at the bottom of the figure.

During the study, a lipid profile was investigated (triglycerides, total cholesterol, LDL, and HDL) in the serum at the end of the treatment. Triglycerides showed a statistically significant reduction in all groups when compared with the HFD group, as shown in Figure 10A. The conventional and nano-formulated teas had no effect on total cholesterol, LDL, or HDL between different groups, as shown in Figure 10B–D.

Although there was no increase in insulin levels in the rat serum, there were differences in the rats’ serum glucose levels; the serum glucose levels at the end of the treatment were significantly decreased in all treated groups except for the nano-formulated low-dose group (100 mg/kg). The conventional low-dose (100 mg/kg) group, both high-dose (300 mg/kg) groups, and the orlistat group showed significant decreases when compared with both the HFD (*p* = 0.0095, 0.0009, 0.02, and 0.03, respectively) and stop diet groups (*p* = 0.001, *p* < 0.0001, 0.003, and 0.003, respectively), as shown in Figure 10E.

The 5 weeks use of the herbal treatment showed no effect on creatinine, ALT, or AST (as a known test for both kidney and liver functions), as shown in Figure 10F–H, respectively.

### 3.9. Herbal Treatment with Conventional and Nano-Formulated Tea Managed to Control Adipocytokines and Anti-Inflammatory Values

**Figure 11:**
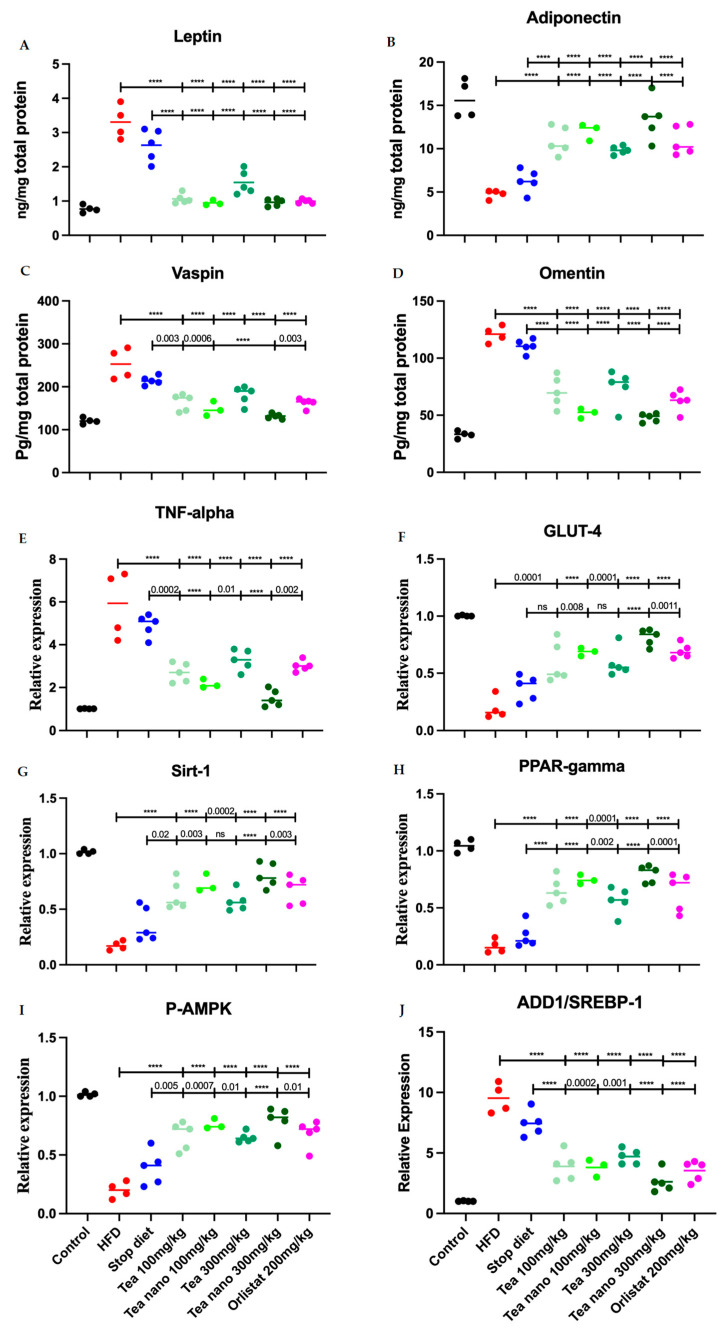
Effects of conventional and nano-tea mixture extracts on (**A**) leptin, (**B**) adiponectin, (**C**) vaspin, (**D**) omentin, (**E**) TNF-α, (**F**) GLUT-4, (**G**) Sirt-1, (**H**) PPAR-gamma, (**I**) P-AMPK, and (**J**) ADD1/SREBP-1. Data represented as scatter plots; significant at *p* < 0.05 are represented as **** (*p* < 0.0001). Different colors are indicating different groups as shown at the bottom of the figure.

Leptin, the peptide hormone, was significantly increased in the adipose tissue of both the non-treated groups (HFD and stop diet) when compared with the control group. While all the treated groups showed a significant reduction in leptin levels when compared with the HFD (*p* < 0.0001) and stop diet (*p* < 0.0001) groups, as shown in Figure 11A.

The nano-formulated tea mixture in both doses showed no differences in adiponectin levels in the adipose tissue when compared with the control group. All other groups, treated and non-treated, when compared with the control group, showed a significant reduction in the adipose tissue peptide adiponectin. The adiponectin levels of the treated groups were significantly increased when compared with the HFD and stop diet groups, as shown in Figure 11B.

The adipocytokine vaspin, which increases with obesity, was significantly reduced in the adipose tissue of the treated rat groups (conventional and nano-formulated (100 mg/kg); conventional and nano-formulated (300 mg/kg)), as well as orlistat group when compared with the HFD group (*p* < 0.0001 in all groups). The nano-formulated tea mixture in both doses showed no differences in vaspin levels in adipose tissue when compared with the control group, while HFD, stop diet, low and high doses of conventional tea mixtures, and orlistat showed significant increases in its levels when compared with the control group (*p* = <0.0001, <0.0001, 0.03, 0.0008, and 0.04, respectively). All herbal treatment groups, except for the conventional high-dose (300 mg/kg) and orlistat groups, showed a significant reduction in vaspin levels in adipose tissue when compared with the stop diet group, as shown in Figure 11C.

The nano-formulated tea mixture in both doses (100 and 300 mg/kg) showed no differences in omentin levels in the adipose tissue when compared with the control group, but all the other groups showed a significantly increased level when with to the control group. The treatment with both the conventional and nano-formulated tea mixtures in both doses, as well as the orlistat group, managed to reduce the omentin levels in the rats’ adipose tissue when compared with the HFD and stop diet groups, as shown in Figure 11D.

### 3.10. Herbal Treatments with Conventional and Nano-Formulated Tea Managed to Control the Expression Levels of TNF-α, GLUT-4, Sirt-1, PPARγ, P-AMPK, and ADD1/SREP-1c

The expression level of inflammatory cytokine TNF-α was measured in the adipose tissue of the experimental rats. As with adipocytokine, only the nano-treated groups restored the expression levels of TNF-α near the control levels. Both the conventional and nano-formulated tea mixture groups showed significantly decreased levels of TNF-α in comparison with both the HFD and stop diet groups, as illustrated in Figure 11E.

The expression of GLUT-4 is essential in controlling normal blood glucose levels. The reduction in its expression in the adipose tissue leads to an imbalance of serum glucose levels. The expression of GLUT-4 was significantly reduced in all groups when compared with the control group, except for the high-dose nano-formulated tea (300 mg/kg). However, all the treated groups showed a significant elevation in GLUT-4 expression in the adipose tissue when compared with the non-treated groups (HFD and stop diet) as shown in Figure 11F.

The downregulation of the Sirt-1 gene is associated with many diseases, including type II diabetes and obesity. The detection of the Sirt-1 gene in the adipose tissue of the experimental rats revealed that the high-dose nano-formulated tea (300 mg/kg) showed a statistically non-significant difference when compared with the control group, while both the non-treated and treated groups showed a statistically significant downregulation of Sirt-1 gene expression when compared with the control group. Consistent with the above results, the treated groups showed significant upregulation of the Sirt-1 gene in adipose tissue when compared with the non-treated groups (HFD and stop diet) as shown in Figure 11G.

The PPARγ gene, one of the main regulators of adipogenesis, was statistically significantly upregulated in the adipose tissue of all the treated groups when compared with the non-treated groups (HFD and stop diet). All the groups, treated and non-treated, showed significant downregulation when compared with the control group, as shown in Figure 11H.

P-AMPK expression was downregulated in the adipose tissue of the obese rats in all the groups when compared with the control group. The treatments with both the conventional and nano-formulated teas, as well as orlistat, managed to upregulate P-AMPK significantly when compared with the non-treated groups (HFD and stop diet) as illustrated in Figure 11I.

The expression of ADD1/SREP-1c in liver tissue was significantly upregulated when compared with the control group in both the treated and non-treated groups, except for when using high-dose nano-formulated tea (300 mg/kg). However, treatment with the herbal tea mixture in both formulations and doses, as well as orlistat, managed to down-regulate the gene expression when compared with the non-treated groups (HFD and stop diet), as demonstrated in Figure 11J.

## 4. Discussion

In the current study, the in vitro lipase inhibition assay demonstrated more effective inhibition activity in pancreatic lipase for the tea mixture that had an IC_50_ value lower than that of orlistat and each tea alone. On the other hand, orlistat showed the smallest inhibitory effect on pancreatic lipase; therefore, the tea mixture was proven to be superior to orlistat in lowering serum TG levels.

In addition, the *α*-amylase inhibition assay also showed the effectiveness of the mixture when compared with each tea alone, or even to the standard, acarbose. Thus, the present in vivo study was concerned with investigating the anti-obesity effect of a herbal tea mixture containing white tea, green tea, and oolong tea, both as tea extracts and nano-formulations, on high-fat-diet-induced obesity in rats by targeting a molecular mechanism.

A high-energy-diet strategy has been widely used in animal experiments to induce fat deposition, overweight, and obesity [37]. The final body weight after conventional and nano-formulated high doses of tea mixture substantially decreased compared with the HFD group. The weights of adipose tissue after the high doses (300 mg/kg) for both the conventional and nano-formulated groups, as well as the Orlistat group, were significantly lower than the HFD group, with the nanoparticle formulation having a more significant reduction when compared with the tea mixture extract and orlistat, confirming that the herbal tea mixture successfully suppressed the accumulation of fat in rats. Similar results were also observed in several studies revealing that tea mixtures have a beneficial reducing effect on relative liver and epididymal adipose weights, with varying degrees of suppression [38,39]. No studies have shown the combination’s effect, but as our results reveal, an increased dose of the tea mixture and tea nanoparticles showed higher suppression regarding fat accumulations.

The main component of dyslipidemia, triglyceride (TG), is associated with obesity. The serum TG levels results were significantly decreased in all groups compared with the HFD group. Our study demonstrated an increase in lipid excretion after the administration of a mixture of these teas. A decrease in the lipids absorbed, rather than a decrease in hepatic uptake or the production of endogenous lipids, was suggested by other studies as the reason behind the reduction in serum TG [40]. Like our findings, a study by Jang and Choung (2013) showed that the consumption of tea catechin resulted in a significant reduction in serum and liver TG and free fatty acids levels to near normal, owing to the suppression of body fat accumulation in a dose-dependent manner [37]. Another study that also supported our deduction that tea extracts, specifically, white tea extracts, can reduce serum TG secondary to a high-fat diet was that of Teixeira et al. (2012) [40].

There was no significant difference in HDL-C, LDL-C, or TC levels in our rat model before or after the initiation of treatment. These results are in accordance with those of Fardet et al. (2008) and Maki et al. (2009), which showed no significant effect on blood cholesterol after supplementation with white tea and green tea, respectively [41,42].

The blood glucose level was significantly decreased in the treatment groups when compared with the control, HFD, and stop diet groups. This is because of the catechins present in the tea mixture, which are purported to enhance GLUT-4 expression in the tissue, consequently increasing glucose uptake, leading to a decrease in serum glucose levels. According to the study by Kim and Kim (2013), epigallocatechin gallate (EGCG), which is the main catechin present in green tea, inhibits the proliferation of adipocytes and their differentiation in 3T3-L1 cells, which increases fat oxidation, and subsequently, GLUT-4 expression in adipose tissue increases, leading to an increase in glucose uptake [43]. Another study by Liu et al. (2019) demonstrated that the catechins in green tea and oolong tea regulate glucose levels [44].

The ALT and AST effects were negligible, which shows that the tea mixture and tea nano-formulation do not affect liver function. Also, the creatinine level was unaffected, demonstrating that the tea mixture and tea nano-formulation did not affect kidney function.

To find a pharmacological target for limiting obesity and its pathophysiological repercussions, the biochemical and molecular pathways that adjust metabolic homeostasis were investigated. It has been shown that the signaling molecules derived from adipose tissue play the most significant role in regulating metabolic homeostasis. Furthermore, adipose tissue secretes leptin, adiponectin, and other adipokines to regulate feeding and fasting. During fasting, adipose tissue increases lipolysis, so it releases non-esterified fatty acids. Thus, leptin decreases in the circulation system during fasting, whilst adiponectin increases [45].

These adipokines have been shown to have paracrine and endocrine functions. The overexpression of adiponectin in 3T3-L1 cells increases adipogenesis and lipid storage. Adiponectin has demonstrated an improvement in insulin sensitivity and maintained a healthy expansion of adipose tissue while also preventing the ectopic accumulation of lipids. Adiponectin is inversely proportional to the amount of body fat a person has. Leptin has a vital role in controlling the intake of food; energy expenditure; and, consequently, body weight via its effect on the hypothalamus. Moreover, leptin’s circulating levels have been found to be directly proportional to fat mass, so its receptors are profusely expressed in numerous tissues, especially in adipocytes [45].

In this study, there was a significant decrease in the serum leptin levels in the tea treatment groups when compared with the HFD, stop diet, and orlistat groups. In addition, there was a remarkable increase in the serum adiponectin levels in the tea treatment groups when compared with the HFD, stop diet, and orlistat groups.

According to the current study, the tea mixture led to a decrease in leptin levels because of a decrease in the amount of body fat. Moreover, the tea mixture increased adiponectin levels because of an increase in the gene expression of adiponectin and a decrease in the amount of body fat. According to the study by Essex and Mosawy (2014), the consumption of green tea decreases obesity by lowering leptin levels and via its effect on the hypothalamus, which is in agreement with our study [46]. Also, green tea has shown a higher resting energy expenditure [47]. There was another study by Zheng et al. (2004) that is in agreement with ours that has shown that green tea reduces leptin levels and adipose weight [48]. In addition, the study by Essex and Mosawy (2014) is in accordance with the present study; green tea polyphenols reduce visceral fat stores, which subsequently leads to an increase in the adiponectin gene transcription in the visceral adipose tissue and serum. Also, it reduces the phosphorylation of PPAR-γ, whereas the gene expression of adiponectin increases [46].

**Figure 12:**
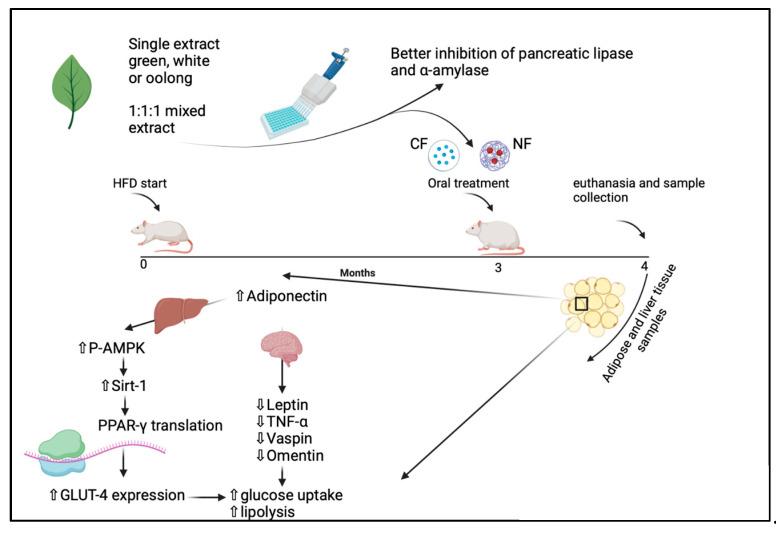
Effects of conventional and nano-tea mixture extracts on molecular pathways in rats with high-fat-diet-induced obesity. This figure was created with BioRender.com.

In adipose tissue, coordinated and balanced activity in the numerous genes that encode the enzymes and proteins that are responsible for lipogenesis, food intake, glucose metabolism, fat storage, and lipolysis is required for metabolic homeostasis, and if an energy excess or an imbalance occurs, it can lead to obesity [49]. To gain insight into the molecular mechanism underlying the tea mixture’s anti-obesity effects, the expressions of genes that are involved in lipid metabolism and obesity, such as pAMPK, Sirt-1, PPARγ, TNFα, and ADD1/SREBP-1c, were studied and demonstrated (Figure 12).

The treatment with nano-formulated tea (300 mg/kg) managed to upregulate P-AMPK, Sirt-1, and GLUT-4 significantly when compared with the non-treated groups (HFD and stop diet). This is in agreement with a study carried out by Manna et al., 2017, in which vitamin D upregulated glucose uptake through the Sirt-1/AMPK/IRS1/GLUT4 cascade in high-glucose-treated 3T3L1 adipocytes [50].

Important insulin-independent signaling molecules like pAMPK and Sirt-1 promote insulin sensitivity and glucose metabolism, where they have been shown to affect insulin resistance-related variables in T2DM. Additionally, AMPK and Sirt-1 can interact and activate one another to establish an AMPK-Sirt-1 cycle, which connects the cell’s energy to its redox state [51]. Moreover, adipogenesis is regulated by p-AMPK and Sirt-1, often called the master metabolic regulators, which work by inhibiting the expression of genes that regulate the differentiation of adipogenesis and the accumulation of triglycerides in 3T3-L1 cells [17]. GLUT4 plays a crucial role in regulating the metabolism of glucose and the maintenance of glucose homeostasis in the body.

The nuclear receptor PPARγ, known as a master transcriptional factor, plays a major role in regulating glucose metabolism and fatty acid storage where it elicits the expression of genes involved in free fatty acid uptake and subsequent triglyceride synthesis, leading to an increase in adipose tissue capacity for the storage of lipids [52]. It is known for its anti-obesity capability and insulin sensitization in adipocytes [53].

The expression of the critical lipogenic transcription factor ADD1/SREBP-1c, which is linked to adipogenesis, occurs in adipose tissue and the liver; this transcription factor has a significant role in the differentiation of adipocytes and the regulation of lipid metabolism by regulating triglyceride synthesis and uptake and the synthesis of fatty acids. Throughout the differentiation process, the levels of ADD1/SREBP-1c mRNA elevate in the pre-adipocytes, and PPARγ expression is triggered by this transcription factor [17].

There was a significant decrease in the mRNA expressions of Sirt-1, p-AMPK, and PPARγ and a significant increase in the mRNA expressions of TNFα and ADD1/SREBP-1c in the HFD group when compared with the control group’s expression levels, as well as all the treated groups. These results suggest that the herbal tea mixture may reduce fat accumulation and weight gain by suppressing the mRNA expression levels of genes that are involved, to a great extent, in fatty acid and triglyceride synthesis, increasing the expression of genes that have anti-obesity effects and a reduction in fat mass, possibly contributing to the lowered TNFα levels because of the decreased inflammation that happens secondary to obesity and the subsequent decrease in oxidative stress. Consistent with these findings, a study by Tian et al. (2013) revealed that the reduction in fat deposits was caused by the administration of green tea polyphenols, which induced and increased the extracellular signal-regulated kinase (ERK) 1/2-PPAR-γ-adiponectin pathway [16]. Moreover, there was an increase in PPARγ mRNA levels that has been linked to the increase in circulating adiponectin in previous studies [54,55], which is in agreement with our results. In addition, these results were supported by the findings of other studies, such as the decreased levels of ADD1/SREBP-1c and TNFα genes and the increased expression of the Sirt-1 gene via the administration of oolong and white teas [17,37,56].

## 5. Conclusions

To conclude, our study showed that the tea mixture has a remarkable effect in managing obesity, specifically, the nano-formulation of the tea mixture, given that it works on both the biochemical and molecular parameters of obesity. This is because of the tea mixture’s ability to restore several adipokines; for instance, it increases the levels of adiponectin and reduces those of leptin. Our study shows that the tea mixture significantly decreased the serum’s glucose, triglycerides, and VLDL. Furthermore, the tea mixture had a significant effect on the mRNA expression of p-AMPK, Sirt-1, TNF-α, ADD1/SREBP-1c, and PPAR-γ, as it increased the mRNA expression of p-AMPK, Sirt-1, and PPAR-γ, all of which induce lipolysis, while also decreasing the mRNA expressions of TNF-α and ADD1/SREBP-1c, inhibiting the inflammation associated with obesity and adipogenesis. In addition, the tea mixture showed a higher weight-reducing effect than orlistat, the standard anti-obesity drug used. Thus, our study suggests that the tea mixture can reduce the body mass index and possibly treat obesity in humans. Further clinical studies are indispensable to proof these results.

## Data Availability

The data presented in this study are available on request from the corresponding author. The data are not publicly available due to that the dataset on which this paper is based is too large to be retained or publicly archived with available resources.

## Supplementary Materials

The following supporting information can be downloaded at https://www.mdpi.com/article/10.3390/metabo13070871/s1: Figure S1: Positive electrospray ionization–tandem mass spectrometry (ESI-MS/MS) analysis of caffeoylquinic acid. Figure S2: Positive electrospray ionization–tandem mass spectrometry (ESI-MS/MS) analysis of caffeoylquinic acid isomers. Figure S3: UPLC-MS analysis of kaempferol glycosides from tea. Figure S4: UPLC-MS analysis of catechins from tea. Figure S5: Top metabolites correlated with anti-obesity activity represented by pancreatic lipase inhibition activity. Table S1: Metabolites annotated from tea extracts after UPLC-MS/MS analysis. Reference [57] is cited in the supplementary materials.
