# Supplementary material for: Anti-Obesity Effect of a Tea Mixture Nano-Formulation on Rats Occurs via the Upregulation of AMP-Activated Protein Kinase/Sirtuin-1/Glucose Transporter Type 4 and Peroxisome Proliferator-Activated Receptor Gamma Pathways
Source: Metabolites. 2023 Jul 21;13(7):871. doi: 10.3390/metabo13070871 (PMC10385210; doi:10.3390/metabo13070871)
Supplement: Supplementary file 1 [file metabolites-13-00871-s001.zip › metabolites-2503122-supplementary.pdf]

metabolites

MDPI

Supplementary Materials

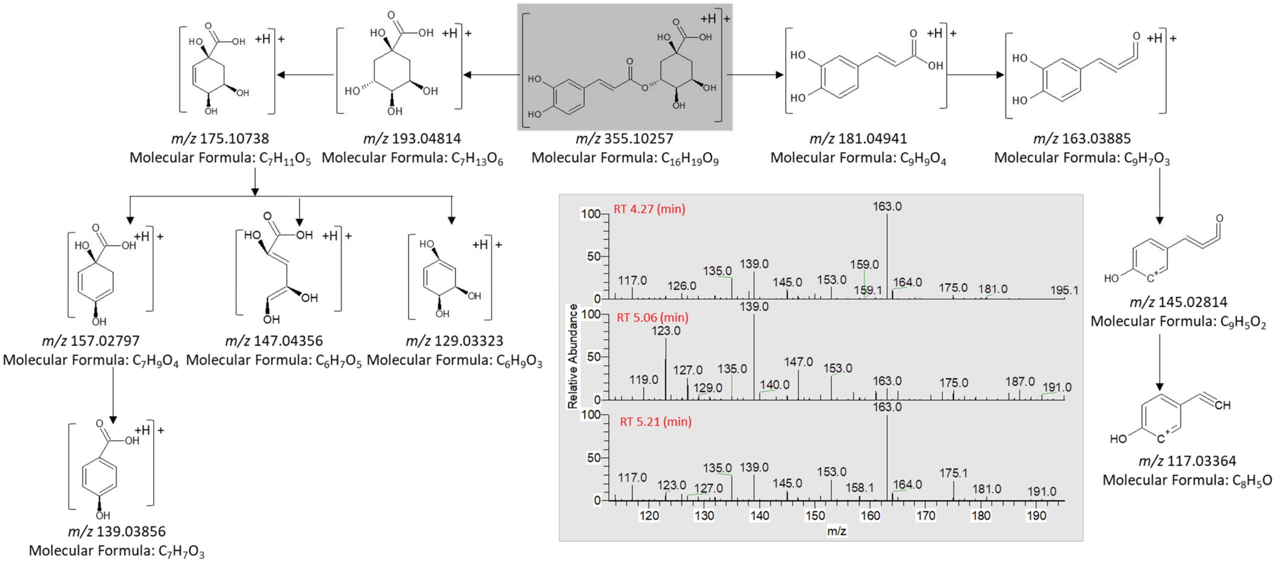

Figure S1. Positive electrospray ionization tandem mass spectrometry (ESI-MS/MS) analysis of caffeoylquinic acid.

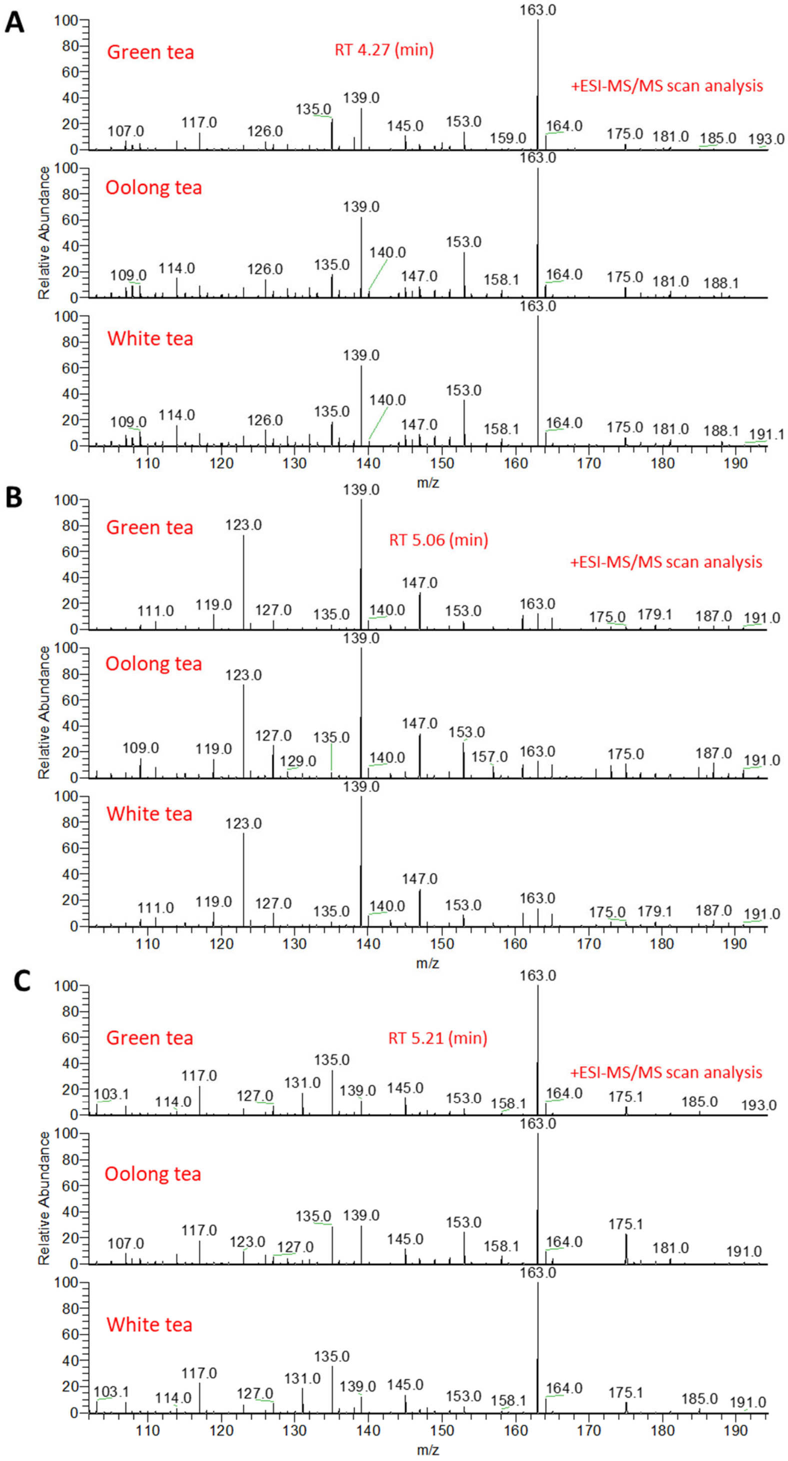

Figure S2. Positive electrospray ionization tandem mass spectrometry (ESI-MS/MS) analysis of caffeoylquinic acid isomers; 5-CQA (A), 3-CQA (B) and 4-CQA (C).

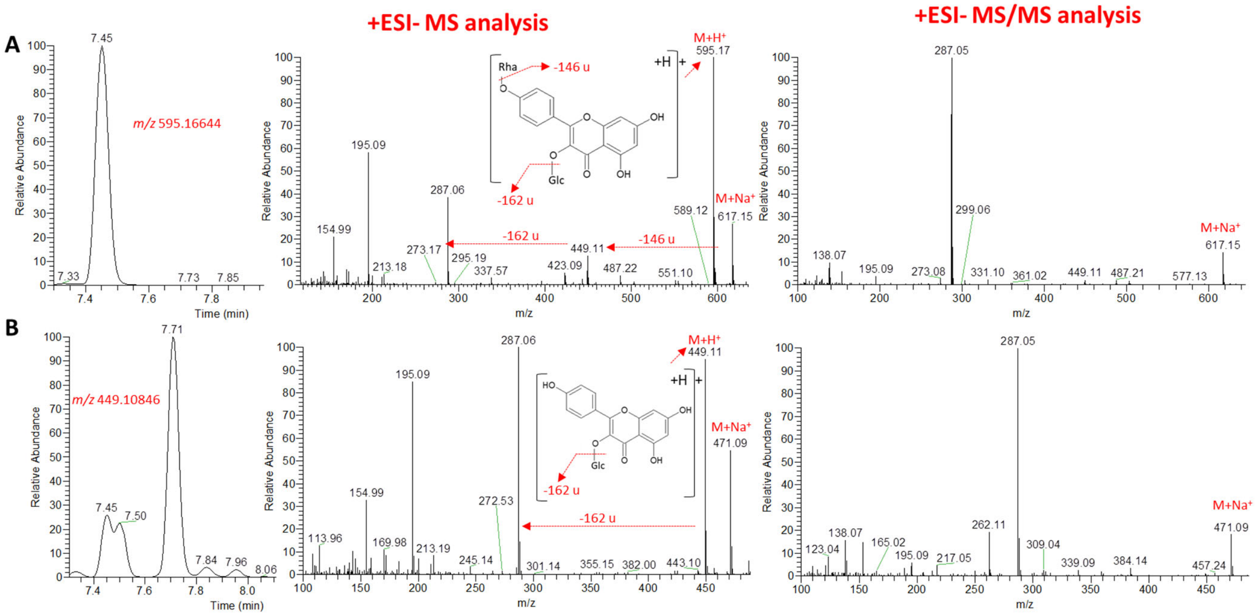

Figure S3. UPLC-MS analysis of kaempferol glycosides from tea. Extracted ion chromatogram (XIC) for the peaks representing kaempferol glucoside rhamnoside (A) and kaempferol glucoside (B) using positive electrospray ionization-mass spectrometry (+ESI-MS).

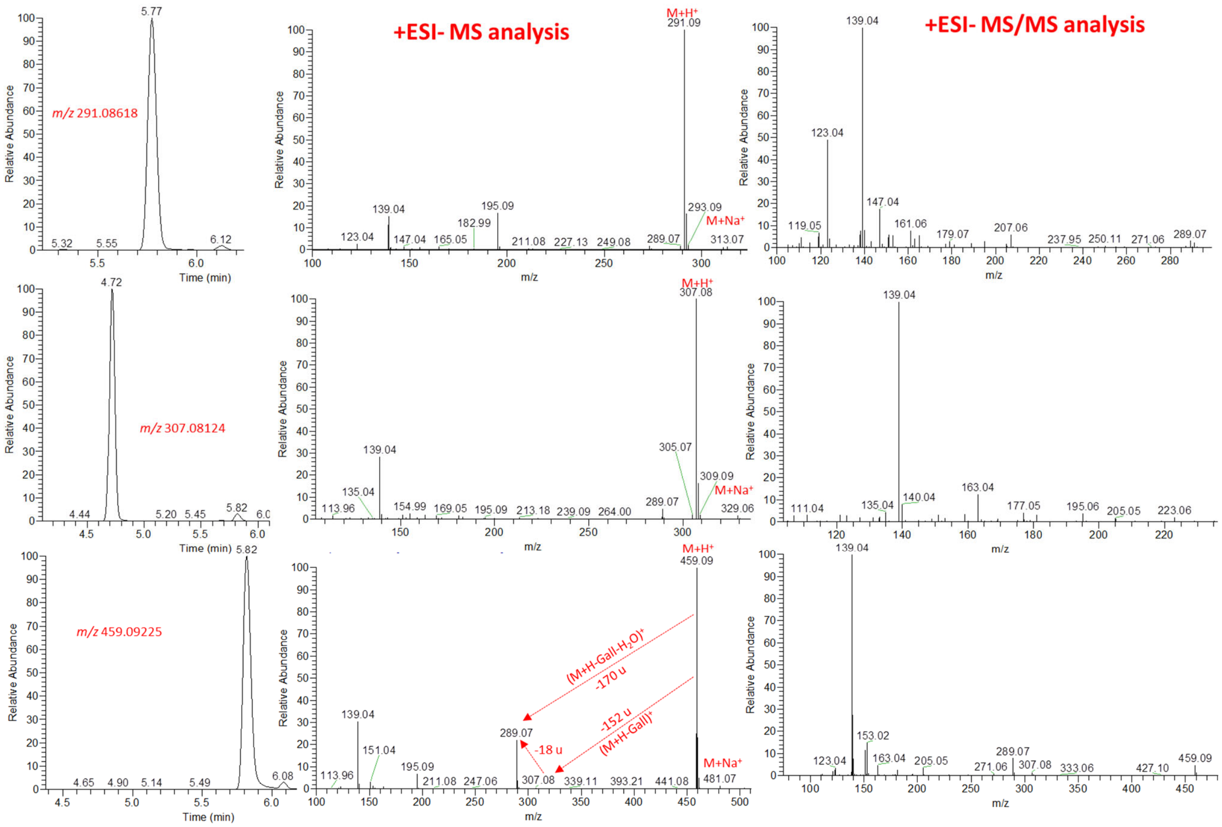

Figure S4. UPLC-MS analysis of catechins from tea. Extracted ion chromatogram (XIC) for the peaks representing catechin (A), gallocatechin (B) and gallocatechin gallate (C) using positive electrospray ionization-mass spectrometry (+ESI-MS).

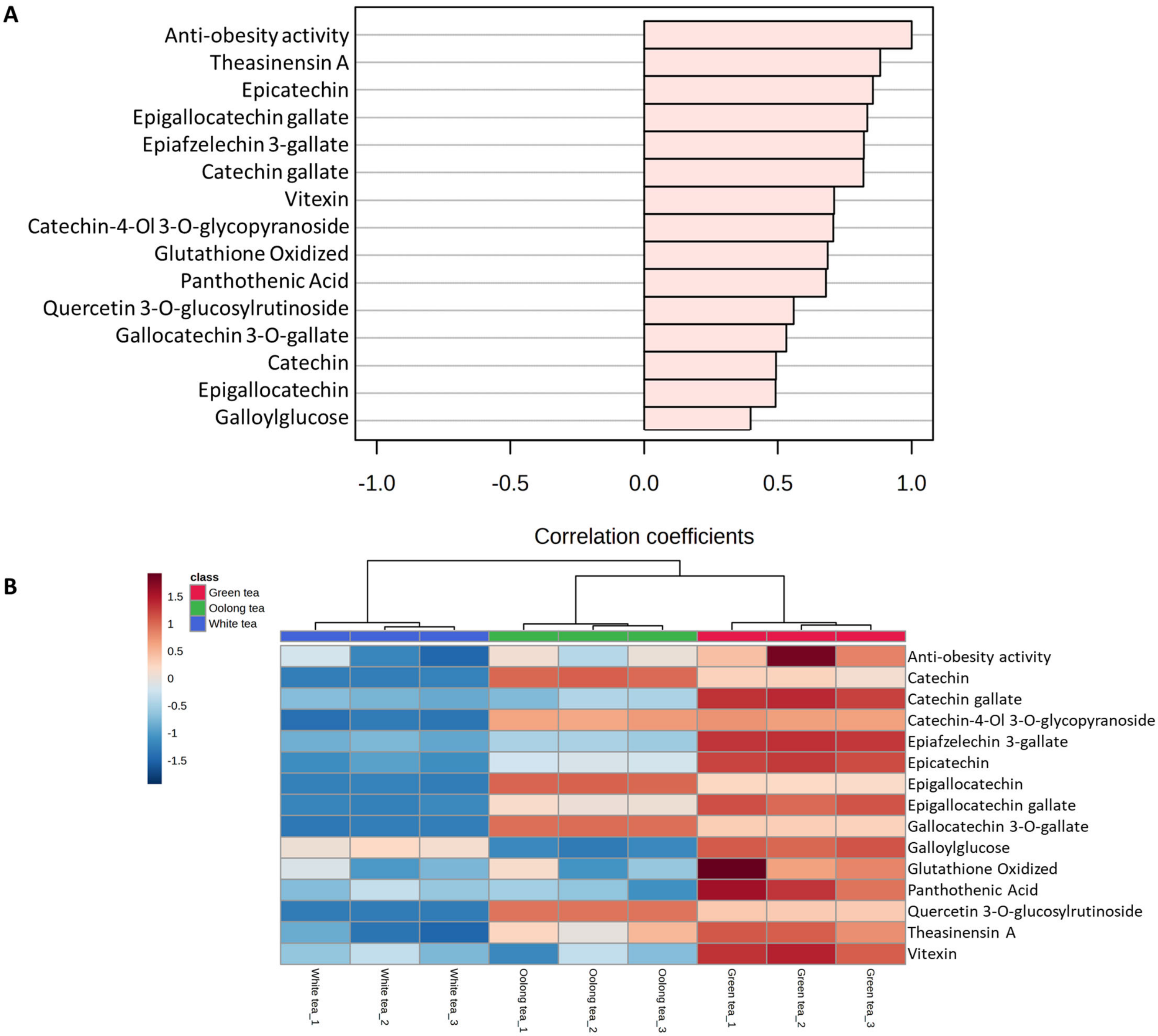

Figure S5. Top metabolites correlated with anti-obesity activity represented by pancreatic lipase inhibition activity. **A.** Pearson's correlation coefficients indicate the relationship between metabolites and pancreatic lipase inhibition activity. **B.** Heat map for the distribution of metabolites correlated with the pancreatic lipase inhibition. The average metabolite abundance from three biological replicates was used for the generation of heat maps.

Table S1. Metabolites annotated from tea extracts after UPLC-MS/MS analysis.

Compound Name	Formula	Detected m/z	Delta (ppm)	RT (min)	Green tea_1	Green tea_2	Green tea_3	Oolong tea_1	Oolong tea_2	Oolong tea_3	White tea_1	White tea_2	White tea_3
4-Caffeoylquinic acid #	C16H18O9	355.1026	0.59	5.2	19281984	19261982	17828852	428209	423013	429595	6195707	6071654	6200066
5-Caffeoylquinic acid #	C16H18O9	355.1025	0.42	4.26	5986542	5597613	5429651	197615	234222	209085	1902277	2078854	1923423
6-Hydroxynicotinic Acid	C6H5NO3	140.0343	0.7	2.43	52765	30512	22351	53567	25273	34818	81628	45310	38939
Apigenin 6-C-glucoside 8-C-arabino-side	C26H28O14	565.1556	0.81	6.29	10710718	10500091	10278104	1.29E+08	1.29E+08	1.22E+08	30977576	31977698	30753866
Caffeine #	C8H10N4O2	195.0876	−0.11	5.26	8.21E+09	7.89E+09	8E+09	3.86E+09	4.02E+09	4.03E+09	7.76E+09	7.69E+09	7.34E+09
Catechin #	C15H14O6	291.0862	−0.35	5.77	4.33E+08	4.29E+08	3.92E+08	6.73E+08	6.97E+08	6.7E+08	1.71E+08	1.7E+08	1.74E+08
Catechin gallate	C22H18O10	443.0974	0.24	6.99	8.15E+08	8.34E+08	7.89E+08	3.87E+08	4.3E+08	4.25E+08	3.9E+08	3.81E+08	3.68E+08
Catechin-4-Ol 3-O-glycopyranoside	C21H24O12	469.1318	−4.79	6.07	26014060	23605056	22989732	22547804	21764732	24679842	1523951	1749850	1638752
Cinnamic acid #	C9H8O2	149.0598	0.52	3.27	4439384	5162491	4628289	250044	284695	284743	4217960	4894406	4163018
Coumaroylquinic acid #	C16H18O8	339.1076	0.47	5.89	25017584	23549514	24765144	2435689	2560064	2466344	10970228	12006367	11399339
Epiafzelechin 3-gallate	C22H18O9	427.1029	1.35	7.84	34319554	35188845	34078953	7603263	7623562	7144264	5745980	6120647	5375483
Epicatechin #	C15H14O6	291.0862	−0.46	5.05	1.8E+08	1.86E+08	1.75E+08	79334750	82252603	80525202	47352807	52534241	47564892
Epicatechin 3-glucoside	C21H24O11	453.139	−0.22	6.52	193357	197226	192699	424804	435406	384198	110764	103835	100021
Epicatechin 3-O-(3-O-methylgallate)	C23H20O10	457.1132	0.52	7.81	539520	448937	497366	21644919	22164645	21691882	141488	157641	130374
Epigallocatechin #	C15H14O7	307.0812	0.03	3.8	44840297	43699803	42427499	1.2E+08	1.21E+08	1.18E+08	7145960	7338307	6922412
Epigallocatechin 3-(4-methyl-gallate)	C23H20O11	473.1079	0.09	6.66	1123138	1004143	1024972	71164248	69333920	71702424	213231	245699	182902
Epigallocatechin gallate #	C22H18O11	459.0923	0.15	5.81	1.07E+09	1.01E+09	1.06E+09	7.22E+08	6.91E+08	6.92E+08	4.2E+08	4.11E+08	4.27E+08
Gallocatechin	C15H14O7	307.0813	0.13	4.72	2.55E+08	2.26E+08	2.38E+08	7.71E+08	8.22E+08	7.73E+08	59715823	64571245	62783061
Gallocatechin 3-O-gallate	C22H18O11	459.0927	1.01	6.08	33175142	32758430	31496218	81761358	82549597	81326080	3463175	3801373	3772607
Galloylglucose	C13H16O10	333.0816	0.03	2.54	1015576	932951	1055180	93136	80624	92689	341232	402125	364913
Glutathione Oxidized #	C20H32N6O12S2	613.1594	0.2	2.2	2017927	734863	850712	495005	188328	268808	391361	192997	236592
Isoleucine #	C6H13NO2	132.1021	1.79	1.92	50843862	52584016	38217915	2110764	2243738	2221396	41220787	49115540	46576073
Kaempferol 3-glucoside-7-rhamno-side	C27H30O15	595.1664	1.17	7.44	37854134	38630012	38273618	1.32E+08	1.35E+08	1.37E+08	1.27E+08	1.26E+08	1.3E+08
Kaempferol 3-O-glucoside	C21H20O11	449.1085	1.38	7.71	19368045	19701707	20257092	51739360	54233437	51447262	42412228	44173552	40936414
Leucine #	C6H13NO2	132.1021	1.56	2.08	69239691	73305707	58877382	2917896	3146124	3079156	44931802	54624694	49369227
Methyl jasmonate	C13H20O3	225.1486	0.17	5.87	130079	95812	104767	1121993	1089949	1128180	296127	325498	317624
Methylxanthine #	C6H7N4O2	168.0632	−5.89	2.83	596926	624114	621791	152810	183441	160836	1437620	3885539	2555870
Myricetin 3-galactoside	C21H20O13	481.0982	1.06	6.42	24947453	24523520	24875727	76706804	81452921	77336779	37695213	39660552	38012826

N-Acetyl-Tryptophane	C13H14N2O3	247.1076	−0.34	7.13	103415	80743	86553	38158	22290	21913	94950	114747	114006
Panthothenic Acid	C9H17NO5	220.118	0.42	3.72	4294532	4023815	3680716	2600323	2527937	2270739	2475101	2741010	2538153
Phenylalanine #	C9H11NO2	166.0864	0.66	3.27	4.73E+08	5.24E+08	4.8E+08	22843567	25824174	23928961	4.36E+08	4.95E+08	4.61E+08
Proline #	C5H9NO2	116.0709	2.28	0.67	132066	66959	139851	112046	176102	144712	75100	181028	151123
Quercetin 3,7-dirhamnoside	C27H30O15	595.1658	0.14	6.61	1489257	1382867	1460600	61588931	61232372	63535543	1057996	975859	919589
Quercetin 3-glucoside #	C21H20O12	465.1034	1.44	7.12	39494536	40134942	39893065	1.2E+08	1.23E+08	1.23E+08	1.27E+08	1.36E+08	1.23E+08
Quercetin 3-O-glucosylrutinoside	C33H40O21	773.214	0.65	6.62	17691032	17017512	17023256	1.49E+08	1.47E+08	1.48E+08	52290	51244	50124
Quercetin- O-rutinoside #	C27H30O16	611.1613	0.97	6.88	61917744	59817456	61602164	2.16E+08	2.14E+08	2.24E+08	2.16E+08	2.17E+08	2.18E+08
Theaflavin	C29H24O12	565.1346	0.94	9.11	2975347	2937889	2866946	712805	746377	753780	6083317	5867731	5800131
Theaflavin 3,3'-digallate	C43H32O20	869.1559	−0.03	9.56	2086328	2495365	2679620	42865	61281	52043	6248837	7407354	7197808
Theaflavin-3-gallate	C36H28O16	717.1453	0.35	9.4	1635946	1655994	1596104	137526	139757	167094	4797835	4611507	4701137
Theasinensin A	C44H34O22	915.16	−1.61	5.82	4308696	4267318	3795647	3117946	2818284	3410687	2047404	1698038	1614575
Theasinensin B	C37H30O18	763.15	−0.61	4.26	2082973	1819919	1812112	558565	534997	536188	4928008	4121515	4248376
Theasinensin C	C30H26O14	611.1396	0.09	2.51	129744	131585	140045	119645	113308	135697	178608	185451	197886
Theobromine #	C7H8N4O2	181.072	−0.31	3.82	7.17E+08	7.06E+08	7.41E+08	51654093	54992955	52755691	6.22E+08	6.63E+08	6.27E+08
Theogallin #	C14H16O10	345.0817	0.21	3.02	5.82E+08	4.65E+08	5.31E+08	33644791	28829873	31062117	3.57E+08	2.85E+08	2.95E+08
Tryptophan #	C11H12N2O2	205.0973	0.51	4.31	1.29E+08	1.22E+08	1.22E+08	17508966	18300352	18349954	62910114	73826182	68671607
Tyramine #	C8H11NO	138.0916	1.54	2.03	20596	9868	102274	25955	16303	32671	22156	33065	32577
Tyrosine #	C9H11NO3	182.0815	1.55	1.92	25842043	26687149	19337812	1746195	1443308	1366197	22169346	25531568	23689902
Uric Acid #	C5H4N4O3	169.0347	−5.16	1.3	21585	26650	34235	32786		29951	27039	32458	31098
Vitexin #	C21H20O10	433.1133	0.9	6.99	5.91E+08	6.02E+08	5.66E+08	3.88E+08	4.48E+08	4.2E+08	4.25E+08	4.48E+08	4.16E+08

This metabolite was detected in the human blood using the public Human Metabolome Database (HMDB) [57].

metabolites

References:

57. Wishart, D.S.; Guo, A.; Oler, E.; Wang, F.; Anjum, A.; Peters, H.; Dizon, R.; Sayeeda, Z.; Tian, S.; Lee, B.L.; Berjanskii, M.; *et. al*. HMDB 5.0: The Human Metabolome Database for 2022. *Nucleic Acids Res.* **2022**, *50*, 622–631.

.
